# Role of the Central Cholinergic Nervous System in Motor and Non-Motor Symptoms of Parkinson’s Disease

**DOI:** 10.2174/011570159X368923250313045859

**Published:** 2025-03-18

**Authors:** Si-Yuan Tian, Xin Cao, Guo-Jin Liu, Ying Zi, Hui-Xian Zhu, Yi-Miao Jiang, Wei-Wei Lou, Xiao-Xia Fang, Ling Shan, Zhan Liu, Qian-Xing Zhuang

**Affiliations:** 1 Department of Physiology, School of Medicine, Nantong University, 19 Qixiu Road, Nantong, Jiangsu, 226001, China;; 2 Department of Neuropsychiatric Disorders, Netherlands Institute for Neuroscience, An Institute of the Royal Netherlands Academy of Arts and Sciences, Meibergdreef 471105 BA, Amsterdam, The Netherlands

**Keywords:** Parkinson’s disease, acetylcholine, cholinergic system, motor symptoms, non-motor symptoms, dopamine agonists

## Abstract

Parkinson’s disease (PD) is a prevalent neurodegenerative disorder that is characterized by both motor and non-motor symptoms. Although dopamine agonists have been demonstrated to be efficacious in the treatment of motor symptoms, their capacity to enhance non-motor symptoms remains constrained. This suggests that additional neurotransmitter systems may be involved in the pathogenesis of PD-related symptoms. The cholinergic nervous system plays a pivotal role in the central nervous system, with various projection systems associated with diverse functions, including but not limited to learning, memory, attention, posture, balance, eye movement control, and adaptation. Nevertheless, the role of the cholinergic nervous system in the motor and non-motor impairments associated with PD remains uncertain. This review elucidates the location, projection, receptors, and effects of central cholinergic systems, as well as their role in both the motor symptoms and non-motor symptoms of PD. Additionally, it examines the crosstalk between cholinergic systems and dopaminergic systems in PD pathology. A deeper comprehension of the fundamental mechanisms of the cholinergic system in PD may facilitate the development of novel therapeutic strategies.

## 1 INTRODUCTION

### 1.1 Parkinson’s Disease

Parkinson’s disease (PD) is a major neurodegenerative disorder and the second most prevalent after Alzheimer’s disease. It poses a significant challenge to global public health [1]. The primary symptoms of PD encompass both motor and non-motor manifestations. The main motor symptoms include resting tremors, muscle rigidity, bradykinesia (slowness of movement), postural instability (an inability to maintain an upright, stable posture), and gait difficulty (difficulty walking due to impaired motor control). Non-motor symptoms involve olfactory disorders, sleep disturbances, autonomic nervous system dysfunction, and cognitive and mental disorders such as depression and apathy [2, 3]. While dopamine has been shown to be an effective treatment for motor symptoms, including bradykinesia and rigidity, its impact on other symptoms remains limited. This suggests that additional neurotransmitter systems may be involved in the expression of PD-related symptoms [4].

Moreover, both human and animal studies have demonstrated that, in addition to dopamine, adenosine, norepinephrine, serotonin, and histamine, acetylcholine is also involved in the pathogenesis of PD and the presentation of its symptoms [2, 5-8]. A substantial body of evidence from numerous studies has demonstrated the significant role of the central cholinergic nervous system in both motor symptoms (such as tremor, postural instability, and gait difficulty) and non-motor symptoms (such as cognitive impairment and dementia, hallucination, depression, emotional apathy, sleep dysfunction, and olfactory dysfunction) of PD [9-16] (Table **1**). Therefore, a comprehensive understanding of the underlying mechanisms of the central cholinergic nervous system in PD development is crucial for facilitating innovative therapeutic strategies aimed at enhancing patient quality of life and prolonging survival.

### 1.2 Cholinergic System Anatomy and Receptors and their Role in PD

**Fig. (1):**
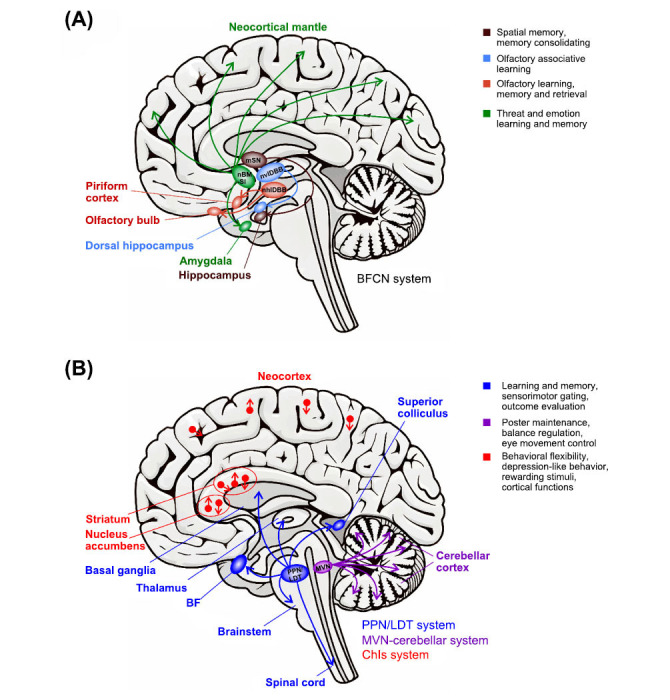
The distribution, projections, and role of the cholinergic system in the central nervous system. (**A**) The composition of the BFCN projection system and its neural projections within the brain. (**B**) The PPN/LDT and MVN-cerebellar cholinergic system, including their projection into the brain and the distribution of cholinergic interneurons (ChIs) in the brain. The red dots depicted in the striatum, nucleus accumbens, and cerebral cortex signify ChIs. In contrast, the red arrows illustrate the connections between these ChIs and other neuronal populations within these nuclei.

The cholinergic nervous system is primarily composed of four subsystems within the central nervous system: the basal forebrain cholinergic nuclei (BFCN) projection system, the pedunculopontine nucleus/laterodorsal tegmental (PPN/LDT) cholinergic projection system, the medial vestibular nucleus (MVN)-cerebellar cholinergic projection system, and the cholinergic interneurons (ChIs) projection system [4, 17, 18] (Fig. **1**). Among these subsystems, the BFCN projection system is the most extensively researched and distinctive. It is located at the rostral aspect of the hypothalamus and on the ventral side of the striatum-globus pallidus complex [18]. This projection system primarily targets the prefrontal cortex, thalamus, and striatum [17, 19, 20]. The BFCN projection system exhibits a parallel organizational structure; however, it is not homogeneously distributed throughout the BF. Instead, these neurons form four discrete clusters known as Ch1-4. These are identified as follows: medial septal nucleus (mSN/Ch1), nucleus of vertical limb of diagonal band of Broca (nvlDBB/Ch2), nucleus of horizontal limb of diagonal band of Broca (nhlDBB/Ch3), and nucleus basalis of Meynert-substantia innominata (nBM-SI/Ch4) [17, 18]. Among these branches, Ch1 and Ch2 project to the hippocampus, while Ch3 projects to the olfactory bulb and piriform cortex; Ch4 is responsible for the majority of projections to the amygdala and neocortical mantle regions. Cholinergic MVN neurons have been observed to innervate portions of the cerebellar cortex, exerting a notable influence on it [18]. The efferent fibers of the PPN/LDT cholinergic projection system are situated within the brainstem, and its boundary exhibits no discernible fiber bundles or other notable anatomical features. The targets of PPN-LDT cholinergic neurons include the basal ganglia, BF, multiple thalamic nuclei, superior colliculus, various brainstem targets, and the spinal cord. ChIs have been identified in the striatum, nucleus accumbens, and neocortex [21], constituting approximately 1-2% of the total number of neurons [22, 23]. These ChIs form dense dendritic structures and primarily project to various neurons in the aforementioned brain regions, including striatal projection neurons, dopamine-releasing terminals, and nearby pyramidal neurons.-Cholinergic projections from the PPN/LDT region and striatal ChIs are of particular importance in the emergence of motor symptoms associated with PD, particularly in motor subtypes that are characterised by postural instability and gait disorders [24].

**Fig. (2):**
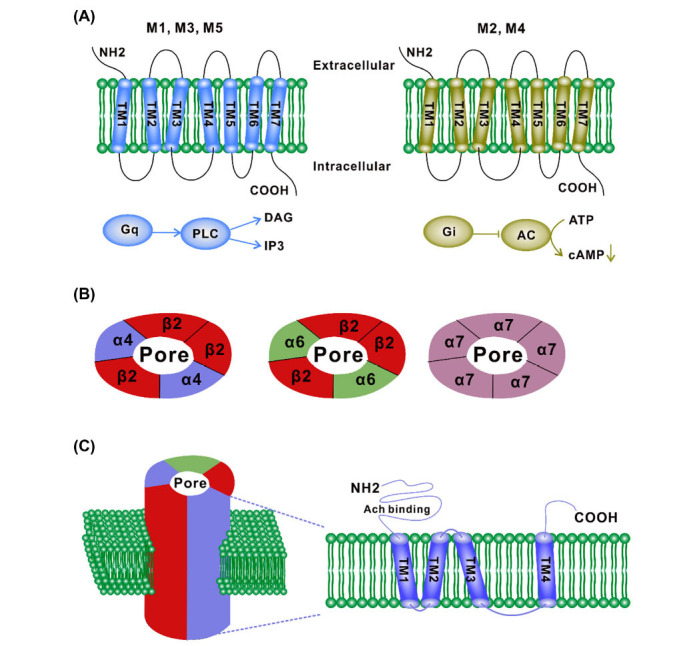
The structural and component characteristics of the AChR. (**A**) Schematic representation of mAChRs illustrates its seven transmembrane domains (TM1-TM7) that traverse the lipid bilayer, along with their primary signaling pathways. M1R, M3R, and M5R are coupled with Gq proteins, whereas M2R and M4R are associated with Gi proteins. (**B**) The illustration depicts representative configurations of nAChR subunits in a pentameric configuration around a cation-permeable pore, which is filled with water. The most prevalent nAChRs in the brain are heterooligomeric α4β2 nAChRs and homo-oligomeric α7 nAChRs. (**C**) The transmembrane topology of nAChR subunits is characterized by a linear structure comprising four transmembrane domains (TM1-TM4) that traverse the lipid bilayer. **Abbreviations**: DAG, diacylglycerol; IP3, inositol triphosphate; AC, adenylyl cyclase; PLC, phospholipase A.

The primary mode of cholinergic neurotransmission is through the action of muscarinic and nicotinic acetylcholine receptors (mAChRs and nAChRs). These receptors are predominantly distributed in postsynaptic sites of cholinergic interneurons and GABAergic neurons in the striatum [2]. They are members of the G protein-coupled receptor family and can be classified into five distinct subtypes: The subtypes are M1R, M2R, M3R, M4R, and M5R (Table **2**). Among these subtypes, M1R, M3R, and M5R are coupled to Gq, while M2R and M4R are coupled to Gi (Fig. **2A**). The excitability of neurons can be increased by acetylcholine receptors coupled with Gq, which is a crucial process in learning and memory. In contrast, Gi-coupled acetylcholine receptors have the capacity to inhibit the excitability of neurons, thereby performing a neural inhibitory function [1, 25]. This regulatory mechanism is beneficial in maintaining the stability and balance of the nervous system. M1R is predominantly expressed in the neocortex, hippocampus, and striatum. Activation of M1R has been demonstrated to enhance the formation of learning and memory. It has been demonstrated in studies that the activation of M1R can achieve this effect by increasing synaptic plasticity and strengthening connections between neurons [26, 27]. Therefore, M1R is considered a crucial receptor in the process of learning and memory. M2R is predominantly expressed in the hippocampus, cortex, BF, and thalamus. Activation of the receptor can result in a number of effects, including neuronal inhibition, analgesia, tremor, and hypothermia. These effects indicate that M2R plays a pivotal role in regulating emotion, pain perception, and motor control [28-30]. Additionally, M2R plays a role in regulating sleep and arousal, which is essential for maintaining normal biological rhythms [31].

M3R is predominantly expressed in the peripheral nervous system, although it is also present in the central nervous system, specifically in the cortex and BF [32, 33]. Similar to M1R, it has been shown to enhance learning and memory abilities. Studies have demonstrated that activation of M3R can achieve this effect by increasing synaptic plasticity and enhancing connections between neurons [34, 35]. Consequently, M3R is considered a pivotal receptor in the process of learning and memory. M4R is predominantly expressed in the striatum, neocortex, hippocampus, and basal ganglia [36]. Of these, the most prevalent is found in the dorsal striatum. M4R has been demonstrated to reduce the release of dopamine in the striatum, thereby regulating the rhythm of motor activity [1]. Moreover, M4R has been shown to possess antipsychotic properties, which can reduce the incidence of psychotic symptoms such as hallucinations and delusions [36]. Finally, M5R is expressed exclusively in dopaminergic neurons. Dopamine is a critical neurotransmitter that plays a pivotal role in regulating emotion, reward, and motor control. Activation of M5R has been demonstrated to increase dopamine release, thereby enhancing the functions in question [10]. Nevertheless, further research is required to elucidate the specific role and regulatory mechanism of M5R. In conclusion, cholinergic neurotransmission plays a pivotal role through the action of muscarinic and nicotinic acetylcholine receptors. These receptors regulate the excitability and inhibition of neurons by coupling with G proteins, thereby affecting a multitude of physiological processes, including learning and memory, emotion regulation, and motor control. The study of these receptors is beneficial in elucidating the function and regulation mechanism of the nervous system, as well as in identifying potential targets for the treatment of related diseases. Nevertheless, the study of these receptors remains an open field of inquiry, with numerous mysteries yet to be resolved. Further research is necessary to elucidate the intricate functions of these receptors.

The nAChR is predominantly situated within the striatum terminal of the dopaminergic neurons in the substantia nigra (SN). It plays a pivotal role in maintaining equilibrium and regulating dopamine transmission in the striatum by modulating the interaction between ChIs and DA terminals [1]. In the human genome, 10 genes encode alpha subunits, 4 genes encode beta subunits, and the remaining 3 genes encode epsilon, delta, and gamma subunits (Figs. **2B** and **C**). The combination of subunits gives rise to a variety of biophysical properties and pentamer structures [10]. These subunits can be composed of numerous types. However, the brain nAChR population is composed of a more limited number of subunit combinations, which are mainly divided into two categories: one is composed of αβ subunits, and the other is α-homo-oligomers [18]. The principal subtypes observed in the striatum are α4β2 and α6β2, along with α7 subunits. Of these, the α4β2 subunit combination is the most prevalent [1]. These α4β2 (potentially including additional subunits) nAChRs are believed to be instrumental in attention processes [10]. In animal experiments, it has been demonstrated that nAChRs containing β2 subunits mediate the protective effect of 6-hydroxytryptamine-induced nigrostriatal injury in rodents [37]. Moreover, β2 subunits have been shown to regulate the expression of induced dyskinesia [38]. Furthermore, studies have indicated that α6 nAChR plays a significant role in dopamine transport, given that it is abundantly expressed within the striatum [1, 39]. The α7 subunit is highly expressed in the hippocampus and hypothalamus and plays a crucial role in non-neuronal tissues, including immune system cells [16, 40]. A variety of experiments, including those utilizing receptor-targeted drugs and gene knockout techniques, have demonstrated that α7 nAchR plays a role in numerous functions, including development, maintenance, survival, synaptic plasticity, neurotransmitter release, and/or the immune response [37, 40].

## 2 CHOLINERGIC SYSTEM ON THE MOTOR SYMPTOMS OF PD

### 2.1 Tremor

Tremor, defined as an involuntary, rhythmic, and alternating movement of body parts, is a well-documented symptom of PD [41]. Two primary manifestations of tremor in PD have been identified: action tremor and resting tremor [42, 43]. In recent years, significant advancements have been made in the study of tremor in PD, particularly in the two phenotypes of resting tremor: dopamine-reactive tremor and dopamine-resistant tremor [44]. These two types of tremors represent discrete phenomena observed in patients with PD at rest. The mechanisms underlying these two tremors may be related to the interaction between the dopaminergic and cholinergic systems in the brain [45]. Dopamine is known to be a neurotransmitter that plays an important role in the brain, including the regulation of motor control, and a reduction in dopamine levels may contribute to the development of PD [3, 5]. On the other hand, ACh is another neurotransmitter that plays an important role in the brain, including the regulation of motor control [18]. Studies have found that cholinergic junctions can distinguish between dopamine-reactive tremor and dopamine-resistant tremor more effectively than reactive dopaminergic junctions [9]. This discovery offers a novel perspective for elucidating the mechanism of tremor in PD. Koganemaru *et al*. conducted an experiment to investigate the impact of dopamine agonists on mice with tacrine-induced tremor [46]. The findings indicated that the tremor was precipitated by tacrine injection, and the vibration level of mandibular movement exhibited a positive correlation with the acetylcholine level in the striatum. These findings reinforce the pivotal role of the cholinergic system in the tremors associated with PD. Moreover, clinical studies have demonstrated that patients with PD who receive cholinesterase inhibitors also experience an exacerbation of tremors [47-49]. All these findings indicate that the cholinergic system plays a crucial role in the pathogenesis of tremors, potentially due to its influence on the dopaminergic system in the brain, which in turn affects tremor occurrence in PD.

In conclusion, the occurrence of tremor in PD is closely related to dopamine and acetylcholine. The contribution of the cholinergic system to the aetiology of tremors cannot be discounted [9, 50]. Further research is required to elucidate the specific mechanism of the cholinergic system in PD tremor. This will facilitate the development of novel approaches and techniques for the management of PD. For instance, new treatment strategies may be identified by studying the impact of the cholinergic system on the dopaminergic system and by exploring methods for alleviating tremor symptoms in PD through the regulation of the cholinergic system [9, 10, 45]. Moreover, genetic variations in individuals with PD can be investigated to determine their impact on the functions of the cholinergic and dopaminergic systems, as well as the occurrence of tremors in PD [51]. This may provide additional therapeutic targets for the development of more effective therapeutic methods.

### 2.2 Postural Instability and Gait Difficulty

Advanced PD is associated with a range of motor symptoms, including disabling postural instability and gait difficulty. These symptoms include postural imbalance, falls, and gait freezing [52]. Previously, it was assumed that postural instability was primarily associated with the decline in striatal dopaminergic innervation. However, recent findings have revealed that cholinergic nerves play a pivotal role in the pathogenesis of postural instability [10, 53]. A voxel-based analysis demonstrated that the striatum and thalamus are the primary regions associated with falls and gait freezing in patients with PD [54]. The striatum exerts control over goal-oriented movements and habits through both direct and indirect pathways. The direct pathway primarily projects to the entopeduncular nucleus and the substantia nigra reticulum (SNr), exerting direct control over these interface nuclei [3, 5, 55]. In contrast, the indirect pathway exerts its influence on these structures indirectly through the action of GABAergic neurons in the globus pallidus external [56, 57]. In the indirect pathway, the M1 receptor and the D2 receptor are co-expressed [58], while the M4 receptor and the D1 receptor are co-expressed in the direct pathway [59, 60]. The activation of the M4 receptor has been demonstrated to reduce glutamatergic activity in the striatum, thereby inhibiting the discharge of medium spiny neurons (MSNs). This process has been shown to result in a decrease in dopamine released by dopaminergic terminals in the SNc [61, 62]. Conversely, the activation of the M1 receptor has been observed to enhance the sensitivity to glutamate input, thereby increasing the discharge of MSNs [63, 64]. In an experiment conducted by Tanimura and colleagues, a Cre mouse model was used to demonstrate that ChIs play a role in amplifying the indirect pathway's response to axonal stimulation of the parafascicular nucleus (PFN) [56]. ChI plays a pivotal role in the striatum, reducing MSNs firing through M4R in the direct pathway and enhancing MSNs excitability through M1R in the indirect pathway. Ultimately, these mechanisms contribute to motor termination [56, 65]. A recent study indicated that in comparison to the “unfrozen,” the density of cholinergic terminals in the striatum, hippocampus, and amygdala of the “frozen” exhibited a significant decrease. This suggests that the loss of cholinergic interneurons in the caudate nucleus plays a pivotal role in the occurrence of gait freezing [21]. These data collectively indicate that the dysfunction of cholinergic interneurons in the striatum represents a common potential defect in the pathophysiology of falls and gait freezing [10]. Secondly, dopamine loss in the striatum and cholinergic afferent disorder in the cortex may also result in the freezing of gait in PD patients. As a result, information regarding the efficacy of gait, posture and movement is lacking in the striatum circuit [18, 66]. This damages the selection and sequencing of motor movements, which in turn leads to slow and reluctant movement or a complete inability to initiate movement [54].

The thalamus serves as a pivotal node within the basal ganglia-thalamic cortex and cerebello-thalamic cortex circuits and plays a critical role in gait regulation [67]. Compared to non-PD patients, cholinergic innervation within the thalamus was found to be significantly reduced in PD patients [68]. A study using AChT showed that the density of thalamic cholinergic terminals was lower in PD patients who had experienced falls than in those who had not, which was consistent with a postmortem study [52]. In a recent study, Karachi and colleagues examined the effects of selective cholinergic injury on the PPN in primates [69]. They observed that this injury can induce a motor syndrome with obvious postural and gait abnormalities. This evidence suggests that gait disturbance and falls in Parkinson's patients may be related to cholinergic PPN dysfunction. Recent studies have demonstrated that the PPN plays a pivotal role in behavioral flexibility through cholinergic output (which inhibits the motor system through the descending junction) and the inhibition of basal ganglia output [70, 71]. Consequently, PPN dysfunction will result in impaired behavioral flexibility and, at the same time, loss of adaptive responsiveness in the natural environment, which will manifest as postural and gait abnormalities and falls. Moreover, cholinergic denervation in the right posterior thalamus has been found to be closely related to falls [72]. This indicates that impaired visual information processing leads to gait problems, and thalamic cholinergic deficiency is also associated with postural reflex impairment [73]. This further confirms the relationship between PPN-thalamic cholinergic denervation and falls [74]. It has also been demonstrated that inadequate integration of attention and motor function of striatal cholinergic interneurons has also been implicated in the pathogenesis of falls [10]. Through pharmacological and functional magnetic resonance imaging studies, Wang *et al*. concluded that the medial geniculate nucleus (MGN) plays a role in PD dyskinesia and found that the connectivity between the left MGN and the left posterior central gyrus was weakened in patients with gait freezing [67]. The MGN, located in the posterior thalamus, serves as a crucial multi-sensory processing relay station. It plays a pivotal role in vestibular sensory processing and multisensory integration in PD and is associated with gait and balance disorders [52]. The cholinergic MGN receives afferents from the PPN-LDT complex [75]. Deletion of cholinergic terminals in the MGN is most strongly associated with each of the three motor characteristics of postural instability and gait difficulties (PIGD). It is closely related to the clinical grading of PIGD severity [52].

Bohnen *et al*. examined the relationship between regional AChT and freezing and falling [54]. He found that the [18F] FEOBV binding decreased in the amygdala and hippocampus of PD patients with freezing gait. This may indicate a disproportionate degeneration of cholinergic projection neurons in the more rostral BF. Similarly, the decreased binding of [18F] FEOBV in the frontal cortex of PD patients with a history of falls may reflect the preferential loss of more cholinergic projection neuron subsets in the caudal BF [54]. Static functional connectivity analysis demonstrated a significant correlation between the severity of motor symptoms and the strength of cholinergic connectivity in the central cingulate region of PD patients. Furthermore, it was found that the progression of PD symptoms is mainly related to cholinergic hyperconnectivity in motor and sensory regions [76]. A substantial body of research has shown that cognitive processes, such as attention, memory, and executive function, are also essential for maintaining balance [77]. Ach is primarily associated with alertness and cognition, particularly executive function and sustained attention [17]. Therefore, Ach may play a role in maintaining balance and gait. The new probe, as demonstrated by Moehle *et al*., revealed that M4 is the primary muscarinic acetylcholine receptor subtype, which is responsible for regulating dopamine signaling and related motor behaviors. Furthermore, M4 activation has been shown to significantly reduce dopamine release, dopamine receptor signaling, and motor function [78].

## 3 CHOLINERGIC SYSTEM ON THE NON-MOTOR SYMPTOMS OF PD

### 3.1 Cognitive Impairment and Dementia

The prevalence of cognitive impairment in PD is six times higher than that of healthy individuals, making it one of the most significant non-motor manifestations of the disease and an essential component of its natural history [79, 80]. Cognitive impairment can greatly reduce the quality of life for patients, with approximately 80% eventually developing dementia [13]. The pathophysiological mechanism of cognitive impairment in PD is complex and multifaceted. It can be broadly explained from four perspectives: firstly, the deposition of α-synuclein in the frontal lobe, temporal association cortex, limbic area, and other brain regions; secondly, the accumulation of Aβ-amyloid and phosphorylated tau protein; thirdly, disturbances in the neurotransmitter system; and finally, genetic factors [11, 80]. Among these, the most significant neurotransmitter system is the disorder of the dopamine system and cholinergic system, which can be caused by the deposition of α-synuclein and Alzheimer's disease [81, 82]. The prevailing theory to explain cognitive impairment in PD is the “dual syndrome” hypothesis [83, 84]. This theory posits that early or mild cognitive impairment may be caused by dopaminergic loss in the frontal striatum, whereas the transition from PD to dementia may be more dependent on non-dopaminergic and cholinergic posterior cortical dysfunction. In light of these considerations, Bohnen *et al*. proposed the “compensatory” theory, which postulates that dopaminergic loss in the frontal striatum may induce a compensatory dependence of cortical cholinergic circuits. Compared to the “dual syndrome” hypothesis, the “compensatory” hypothesis more explicitly emphasizes the interplay between dopaminergic and cholinergic systems [85, 86]. Dopaminergic denervation may result in damage to specific cognitive areas, particularly in the early stages of the disease. However, *in vivo,* imaging, and postmortem evidence suggest that the progressive cognitive decline leading to dementia in PD is associated with deterioration of the cholinergic system in the BF [87]. Consequently, the cholinergic system plays a pivotal role in cognitive impairment in PD. For instance, research has indicated that the deterioration of the cholinergic system is a primary factor in the development of dementia in PD [86]. Additionally, studies have demonstrated that the cholinergic loss observed in PD patients with dementia is more pronounced than that observed in patients without dementia [88-90]. Even in the absence of dementia, the deterioration of the cholinergic system is a significant contributor to cognitive impairment in PD [88, 91].

Studies have demonstrated the pivotal role of the BF, cerebellum, and hippocampus in the cognitive impairment observed in PD patients [13, 92-94]. The BF is a crucial brain region of the brain, responsible for regulating a variety of cognitive functions, including attention, working memory, and decision-making [95]. Research has revealed a significant correlation between the volume or integrity of the BF and the cognitive impairment observed in PD, as evidenced by magnetic resonance imaging (MRI) [96, 97]. Specifically, the loss of structural integrity and connectivity in the Ch1-2 subregion of the BF is related to memory and visuospatial task performance in PD patients, while the Ch3-4 subregion is associated with executive function and broader cognitive performance [96]. These results indicate that BF plays a key role in the occurrence and development of cognitive impairment in PD. Moreover, studies have demonstrated that cortical cholinergic activity and Ch4 cholinergic neurons are significantly diminished in patients with PD and dementia [13, 97, 98]. For instance, Zhang *et al*. employed a combination of clinical and neuropsychological assessment and MRI to evaluate the functional connectivity of each subregion of the BF. This study demonstrated that, in comparison to PD patients with normal cognition and healthy controls, there was a decrease in FC between Ch4 and the frontal, parietal, and occipital cortices in PD patients with mild cognitive impairment [13]. Rong *et al*. employed the same methodology to analyze the correlation between nBM/Ch4 volume and cortical thickness and to compare the correlation coefficients. They concluded that nBM/Ch4 volume has a strong correlation with cortical thinning and that nBM/Ch4 volume loss may play an important role in PD cognitive impairment [99]. These findings collectively suggest an imbalance between BF and projection, supporting the loss of Ch4 choline neurons. As a result, the expansion of NBM microstructural defects in PD patients signifies cognitive impairment [100]. However, some studies have indicated that BF deterioration is not necessarily associated with significant cognitive impairment. This supports the hypothesis that cognitive decline only occurs when structural changes affect a wider cerebral region [101]. Therefore, the initial presence of Ch4 atrophy in PD patients without cognitive impairment may indicate future development of dementia.

Studies using single-photon emission computed tomography (SPECT) and PET have shown that AChE activity is more extensive and significantly reduced in patients with PD and dementia compared to those without dementia [102]. Moreover, positron tomography has revealed a significant decrease in AChE activity in the cerebral cortex [103]. Research has also indicated that the substantial loss and decrease of cholinergic neurons and various subtypes of nAChR are closely associated with the progression of cognitive decline [25]. For instance, α7 nAChR is highly expressed in the hippocampus, which is particularly affected by cognitive impairment [16]. In addition, the extensive loss of ACh in the cortex is due to the extensive loss of cholinergic neurons, which is also evident in Parkinson's dementia brain [104, 105]. In conclusion, BF and the cerebellum play a pivotal role in the cognitive impairment of PD. The abnormal structure and function of BF and the loss of Ch4 cholinergic neurons may contribute to cognitive impairment in PD patients. Furthermore, dopaminergic and cholinergic projections in the cerebellum may also be involved in the development of cognitive impairment in PD. Future research is needed to further explore the specific role of these mechanisms and how to improve the cognitive function in PD patients by intervening in these mechanisms.

### 3.2 Hallucination

Hallucination is a common non-motor symptom in PD patients, with prevalence rates ranging from 8% to 40%. This symptom has been identified as a risk factor for dementia and increased mortality [106]. Previously, the occurrence of hallucinations was thought to be related to dopamine imbalance [107]. However, recent studies have demonstrated that the cholinergic system also plays a direct role in the development of hallucinations [86, 102, 108]. Firstly, the most common cause of hallucinations in both PD and non-PD patients is the use of anticholinergic medications, particularly visual hallucinations [109, 110]. Anticholinergic drugs inhibit the action of acetylcholine, leading to an imbalance of neurotransmitters and the subsequent occurrence of hallucinations. Moreover, Anticholinergic drugs can increase the prevalence of hallucinations by affecting the excitability and inhibition of neurons. Therefore, when administering anticholinergic drugs to PD patients, it is critical to closely monitor for hallucination symptoms and promptly adjust the dose or implement alternative treatment plans as needed. Secondly, extensive reductions in ChAT activity have been observed in the neocortex of individuals with hallucinogenic Lewy body dementia and PD [110]. ChAT is a pivotal enzyme that facilitates acetylcholine synthesis. A reduction in its activity leads to a reduction in acetylcholine levels, which interferes with the normal transmission of neurotransmitters. The reduction of ChAT in the neocortex may be caused by neuronal injury or metabolic dysfunction, which provides a new explanation for hallucinations in PD patients. Thirdly, the loss of cholinergic neurons in the PPN and thalamic gray matter has been demonstrated to be associated with hallucinations in PD patients [111, 112]. The PPN and thalamus are vital neural structures in the brain that are involved in regulating a variety of physiological functions, including arousal, attention, and emotion. Damage to these regions can result in neurotransmitter imbalances and dysfunction of neural networks, which can subsequently lead to the occurrence of hallucinations [113]. In addition, damage to these areas may also affect the occurrence of other non-motor symptoms, such as cognitive impairment, depression, and anxiety. Fourth, cholinergic inhibition is also an important reason. Shinotoh *et al*. concluded by PET and SPECT that compared with PD patients without hallucinations, PD patients with hallucinations have an obvious cortical cholinergic deficiency [7]. This suggests that damage to the cholinergic system may be an important factor contributing to hallucinations in PD patients.

The thalamic drive hallucinogenic model is currently understood to be based on the alteration of the default mode network function and the downregulation of the thalamoreticular nucleus [107]. PD patients who experience hallucinations tend to have vivid dreams and nightmares [114]. Janzen *et al*. discovered that the gray matter of the PPN and its thalamic target exhibited a reduction in hallucinatory PD patients compared with non-hallucinatory PD patients, as determined by voxel-based morphological measurement technology. They subsequently concluded that the atrophy of the PPN region and its thalamic projection region was more closely associated with hallucinations than the associated cognitive decline when compared with patients with and without dementia [115]. Moreover, the amygdala plays a pivotal role in sensory processing, and the cholinergic nerve is also involved in the innervation of the amygdala. Consequently, when the cholinergic nerve is damaged, the regulatory function of the amygdala is impaired, resulting in the occurrence of hallucinations [12].

In conclusion, the mechanism of hallucination in patients with PD is multifactorial. It involves the use of anticholinergic drugs, a reduction in choline acetyltransferase in the neocortex, the loss of the PPN and thalamic gray matter, and cholinergic inhibition. These factors interact to create a neurotransmitter imbalance and neural network dysfunction, resulting in hallucinations.

### 3.3 Depression

Depression is a prevalent complication of cognitive impairment in PD, which is commonly attributed to an imbalance of serotonin and norepinephrine [116]. However, recent studies have demonstrated that the use of cholinesterase inhibitors can significantly alleviate depressive symptoms in PD patients with dementia [117, 118]. Consequently, the depressive symptoms of PD patients may be partially attributed to the dysfunction of the cholinergic system [119]. Imaging studies have demonstrated that the α4β2 nAChR plays a pivotal role in the pathogenesis of depression [120, 121]. For instance, in a PET study employing the radioligand 2-[18F]fluoro-3-(2[S]-2-azylmethoxy) pyridine, researchers observed a reduction in the binding affinity of the α4β2 nAChR ligand in PD patients with depression [122]. This finding indicates that α4β2 nAChR may play an important role in the occurrence and development of depressive symptoms in patients with PD. Moreover, the loss of cortical cholinergic projections may also result in the onset of depressive symptoms in PD patients [13]. However, this loss of neurons is also the basis of dementia. Therefore, in the early stages of dementia, it is difficult to distinguish the influence of this neuronal degeneration on depressive symptoms [123]. Further research is needed to gain a deeper understanding of this issue. Specifically, studies are needed to examine the relationship between cortical cholinergic neuron loss and depressive symptoms in PD patients, as well as to assess the potential benefits of intervention within the cholinergic system to improve the cognitive and emotional status of patients. In addition, a reduction in the expression of M2/M4 receptors has been observed in the dorsolateral prefrontal cortex of patients with major depression [124, 125]. These receptors play a pivotal role in regulating neurotransmitter release and synaptic plasticity. Dysfunction of these receptors may contribute to the development of depression. Therefore, pharmacological agents targeting these receptors may be an effective way to treat depressive symptoms in patients with PD. In conclusion, depressive symptoms in PD patients may be related to a variety of factors, including cholinergic system dysfunction, abnormal α4β2 nAChR function, and decreased M2/M4 receptor expression.

### 3.4 Emotional Apathy

One of the most common non-motor symptoms in PD patients is emotional apathy [126, 127]. This is characterized by a decreased interest in things and a weakened emotional response. This symptom may be related to the abnormal function of neural networks related to emotional regulation in the brain. Studies have found that the emotional regulation network in the brain includes many brain regions, such as the anterior cingulate gyrus, amygdala, hippocampus, and so on [128, 129]. The interaction between these brain regions is very important in maintaining normal emotional responses. Sperling *et al*. conducted an analysis of brain magnetic resonance imaging and the Frontal Systems Behavior Scales (FrSBe) in PD patients and discovered a significant correlation between Ch4 cholinergic BF gray matter density (GMD) and apathy symptoms in PD patients [130]. The findings of this study provide a novel perspective for elucidating the underlying mechanisms of apathy in PD patients. Moreover, the researchers discovered that degeneration of the Ch4 region in PD patients results in the loss of cholinergic fibers in several cortical and marginal regions [130]. This nerve loss may be caused by the degeneration of dopaminergic neurons, which are an important nerve cell in the brain.

Dopamine plays a variety of physiological roles in the brain, including the regulation of motor, emotional, cognitive, and other functions [131, 132]. Damage to dopaminergic neurons can lead to abnormalities in the functions they regulate. In patients with PD, the degeneration of dopaminergic neurons may result in the loss of cholinergic nerves due to the close interaction between dopamine and choline in the brain [18, 45, 133]. Dopamine can inhibit the activity of cholinergic neurons, while choline can promote the function of dopaminergic neurons. Consequently, when dopaminergic neurons are damaged, it may affect the normal activity of cholinergic neurons, resulting in changes in GMD. This change in GMD may affect the apathy symptoms of PD patients. In patients with PD, the interaction between these affective regulatory brain regions may be affected due to the change in GMD [130, 134]. For instance, the loss of cholinergic fibers may affect the signal transmission between the anterior cingulate cortex and the amygdala, thus influencing the regulation of emotional response, and the loss of cholinergic fibers may also affect the function of the hippocampus, resulting in abnormal regulation of memory and emotion [14]. These factors interact to produce the symptoms of apathy in PD patients. In essence, the research of Sperling *et al*. elucidates the underlying mechanism of apathy symptoms in PD patients [130]. By studying the changes in cholinergic BF GMD in PD patients, insights into the underlying pathophysiology of apathy symptoms may be gained. This offers a new avenue for the diagnosis and treatment of PD and may help to improve the quality of life of people with PD. However, the current state of research on the relationship between GMD changes and apathy symptoms in PD patients is still in its infancy [135, 136]. Further research is needed to substantiate this finding and to determine how it can be used to improve treatment.

### 3.5 Sleep Dysfunction

Rapid eye movement sleep behavior disorder (RBD) is a common occurrence in individuals diagnosed with PD, a condition characterized by the loss of normal skeletal muscle tone and the occurrence of faceful movements during REM sleep [137]. When these symptoms are not accompanied by other indications, the disorder is referred to as idiopathic RBD (iRBD) [138]. It is evident that the occurrence of RBD symptoms in PD patients is associated with relative cholinergic denervation, and acetylcholine levels are associated with the occurrence of sleep dysfunction [15, 139]. Gersel Stokholma *et al*. demonstrated that patients with iRBD have clear neocortical cholinergic denervation as evidenced by PET imaging *in vivo*. Moreover, degeneration of cholinergic neurons in the meynert basal nucleus was observed in postmortem studies of iRBD patients. These findings suggest that iRBD represents an early stage of PD and Lewy body dementia [15]. In addition, Kotagal *et al*. demonstrated that the occurrence of RBD symptoms in PD patients is related to cholinergic denervation in the neocortex, marginal cortex, and thalamus. Additionally, they found that the cholinergic projection system in the pontine tegmentum and BF complex may play a key role in the pathogenesis of RBD in PD patients [140]. Subsequently, it was demonstrated that the activity changes of the PPN and LDT may serve as the foundation for the pathophysiology of RBD. The cholinergic neurons of the PPN and LDT regulate wakefulness, maintain wakefulness, and regulate the initiation of REM sleep [141]. Studies have indicated that the PPN/LDT complex contains cholinergic neurons that are active during REM sleep [142], and α-synuclein may cause excitation and increase calcium influx, subsequently damaging the PPN/LDT and impairing its ability to regulate sleep [141]. In conclusion, the denervation of neocortical cholinergic neurons was significantly increased in RBD patients, and cholinergic neurons in the BF and PPN/LDT systems showed degeneration. These findings suggest that cholinergic drugs may have a potential effect on the treatment of RBD.

### 3.6 Olfactory Dysfunction

Olfactory dysfunction is an early non-motor manifestation of PD that may occur in the prodromal stage of the disease and may precede before the loss of dopaminergic energy in the midbrain [86]. However, recent studies have also demonstrated that the cholinergic system may be involved in the pathophysiological process of olfactory dysfunction in PD patients [143, 144]. Versace and colleagues found that the majority of subjects with overt olfactory dysfunction were accompanied by mild cognitive impairment [144]. Nevertheless, the precise pathophysiological mechanism of olfactory dysfunction in PD remains elusive [21]. The loss of olfactory sensation may be attributed to the demise of dopaminergic neurons in the midbrain. Midbrain dopaminergic neurons play a pivotal role in the regulation of olfactory function. These neurons establish connections with the olfactory bulb and other olfactory-related areas, thereby controlling olfaction [145-147]. It has been proposed that dopaminergic neurons in the midbrain of Parkinson's patients may gradually lose function, resulting in impaired olfactory function. This type of olfactory impairment may manifest as a decreased ability to detect odors or a decreased sensitivity to certain odors [148-150].

Despite increasing evidence that the cholinergic system plays a role in the pathophysiological process of olfactory dysfunction in PD, the precise mechanism remains elusive [21, 15]. Current research has focused on the interaction between dopaminergic and cholinergic neurons in the midbrain. Some studies have postulated that an imbalance between these two neuronal populations in the midbrain may contribute to olfactory dysfunction [16, 151]. For instance, the loss of dopaminergic neurons in the midbrain may result in an abnormal function of cholinergic neurons, thereby affecting olfactory function [16]. Moreover, studies have demonstrated that oxidative stress and inflammatory reactions may be involved in the onset and progression of olfactory dysfunction in PD [152, 153]. Oxidative stress and inflammatory responses may lead to neuronal damage and death, thereby affecting olfactory function. In addition, some studies have found that patients with PD may have an abnormal structure and function of the olfactory pathway, which may also be one of the causes of olfactory dysfunction [154, 155]. To summarize, olfactory dysfunction in PD is a complex pathophysiological process that may involve the interaction of various neurotransmitter systems and multiple pathophysiological mechanisms. Although some progress has been made in the current research, the clear pathophysiological mechanism of olfactory dysfunction in PD still requires further investigation. Future research should focus on elucidating the interaction between dopaminergic and cholinergic neurons in the midbrain, as well as the role of oxidative stress, inflammatory response, and abnormal structure and function of the olfactory pathway in PD olfactory dysfunction.

## CONCLUSION AND FUTURE PERSPECTIVES

As a common neurodegenerative disease, PD has a complex pathogenesis and involves the imbalance of various neurotransmitter systems [3, 5, 51]. In recent years, as research on PD has deepened, the role of the cholinergic system in the pathogenesis of PD has been the subject of increasing attention [10, 18, 25]. This review summarizes the role of the cholinergic system in PD and provides prospective insights into its involvement in tremors, non-motor symptoms, and its relationship with other diseases.

Firstly, the cholinergic system plays an important role in the onset and progression of tremor in PD [9, 48]. The underlying mechanism of tremor in PD may be related to the interaction between the dopaminergic and cholinergic systems in the brain [45, 68, 85]. A reduction in dopamine levels has been associated with the development of PD, while acetylcholine, a crucial neurotransmitter, also plays a role in regulating motor control in the brain. The cholinergic system thus plays a central role in the pathogenesis of PD tremor due to its ability to influence dopaminergic activity within the brain [9, 64]. Moreover, the use of cholinesterase inhibitors has been shown to exacerbate the occurrence of tremor in PD, further supporting the critical role of the cholinergic system in tremor in PD [48, 49].

Secondly, the cholinergic system is also important in the onset and development of non-motor symptoms of PD. These symptoms include cognitive impairment, depression, apathy, sleep disturbance, and so on [13, 130, 140]. The BF, cerebellum, and hippocampus play an important role in cognitive impairment of PD [13, 80]. The abnormal structure and function of the BF and the loss of Ch4 cholinergic neurons may lead to cognitive impairment in PD. In addition, the deterioration of the cholinergic system is the main determinant of PD dementia [117]. Dysfunction of the cholinergic system may also be associated with depressive symptoms in patients with PD who experience depression [120]. Therefore, it is evident that the cholinergic system plays a pivotal role in both the occurrence and development of non-motor symptoms of PD.

Furthermore, it is important to note that there is a noteworthy relationship between the cholinergic system and other diseases. The cholinergic system also plays a critical role in schizophrenia, Lewy body dementia, and other diseases [45, 81]. Dysfunction of this system in schizophrenia may lead to positive and negative symptoms [45]. Consequently, conducting an extensive study on how the cholinergic system functions within PD, as well as other diseases, will help shed light on their pathogenesis while providing new insights for clinical treatment.

In view of the role of the cholinergic system in PD, future research can be conducted on the following aspects:

1. To study in depth the mechanism of the cholinergic system in the occurrence and development of various symptoms such as tremors and non-motor symptoms of PD, and to provide the theoretical basis for the diagnosis and treatment of PD.
2. To explore the interaction between the cholinergic system and other neurotransmitter systems and elucidate the complex mechanism of PD.
3. To study the role of the cholinergic system in different phenotypes of PD and provide personalized treatment for clinical management.
4. To develop drugs for the cholinergic system, such as cholinesterase inhibitors and cholinergic receptor agonists, to improve the symptoms and quality of life of PD patients.
5. To study the role of the cholinergic system in other neurodegenerative diseases and provide new ideas for the treatment of these diseases.

## STUDY LIMITATIONS

Due to the complexity of the central cholinergic nervous system and the extensive distribution of cholinergic receptors within it [40, 125], cholinergic drugs are often associated with side effects during their use, which limits their clinical application. For instance, current anticholinergic medications are primarily suitable for young patients with PD who possess intact cognitive function and can be utilized as short-term adjunctive therapy for dopamine-resistant tremors [41]. Furthermore, the development and clinical implementation of cholinergic drugs have been slow due to concerns regarding insufficient efficacy or safety profiles. At present, rivastigmine is the only drug approved for treating PD-related hallucinations and psychiatric disorders [118]. Consequently, selective regional administration targeting specific brain regions, nuclei, or even particular types of neurons holds promise in minimizing these adverse effects to the greatest extent possible.

In summary, the cholinergic system plays an important role in PD. An in-depth study of the role of the cholinergic system in PD is helpful in elucidating the pathogenesis of PD and providing new ideas and methods for clinical treatment. With the development of research on the cholinergic system, it is reasonable to anticipate that this system will emerge as a promising new target for treating PD.

## Figures and Tables

**Table 1:** The involvement of the central cholinergic nervous system in the manifestation of PD symptoms.

**PD-related ** **Symptoms**	**-**	**Cholinergic Effects**	**References**
Motor symptoms	Tremor	The cholinergic system can affect the dopaminergic system in the brain, which in turn affects the occurrence of tremors.	[9, 46-49]
	Postural instability and gait difficulty	Cholinergic interneuron dysfunction and cholinergic afferent dysfunction are underlying causes of postural instability and gait disturbance.	[10, 18, 66, 72]
Non-motor symptoms	Cognitive impairment and dementia	Loss of BF cholinergic neurons is essential for the development of cognitive impairment and dementia, along with cholinergic denervation in the cerebellum and hippocampus.	[11, 13, 92-94, 156]
	Hallucination	Hallucination is associated with decreased neocortical choline acetyltransferase, loss of PPN and thalamic gray matter, and cholinergic inhibition.	[12, 107, 113, 115]
	Depression	Depressive symptoms in PD patients are associated with cholinergic dysfunction, abnormal α4β2 nAChR function, and decreased M2/M4 receptor expression.	[13, 122, 125]
	Emotional apathy	Ch4 GMD degeneration results in the loss of cholinergic fibers in multiple cortical and marginal regions, contributing to Parkinson's symptoms of apathy.	[14, 130, 134]
	Sleep dysfunction	The occurrence of RBD symptoms in PD patients is associated with cholinergic denervation in neocortical, paracortical, and thalamic areas, and reduction of acetylcholine levels is associated with the occurrence of sleep dysfunction.	[15, 139, 140, 141]
	Olfactory dysfunction	The interaction between dopaminergic and cholinergic neurons in the midbrain may be a contributing factor to olfactory dysfunction.	[16, 151]

**Table 2:** Classification, distribution, and function of central cholinergic receptors.

**AChRs**	**Subtypes**	**Distribution**	**Function**	**References**
mAChRs	M1R	Neocortex, hippocampus, and striatum	Facilitate the formation of learning and memory	[26, 27]
	M2R	Hippocampus, cortex, BF, and thalamus	Regulating emotion, pain perception, motor control, sleep and arousal	[28-31]
	M3R	Across the central nervous system, specifically in the cortex and BF	Enhancing learning and memory	[34, 35]
	M4R	Striatum, neocortex, hippocampus, and basal ganglia	Regulating the rhythm of motor activity, reducing the incidence of psychotic symptoms	[1, 36]
	M5R	Dopaminergic neurons	Increasing dopamine release	[10]
nAChRs	α4β2	Primarily located in the striatum terminal of the dopaminergic substantia nigra	Attention function	[10]
	β2		protection of substantia nigra striatum injury and regulating dyskinesia	[37, 38]
	α6		Dopamine transport	[1, 39]
	α7	Highly expressed in the hippocampus, hypothalamus, and immune system cells	Involving development, maintenance, survival, synaptic plasticity, neurotransmitter release, and/or the immune response	[37, 40]

## LIST OF ABBREVIATIONS

BF: Basal Forebrain
BFCN: Basal Forebrain Cholinergic Nuclei
ChIs: Cholinergic Interneurons
FrSBe: Frontal Systems Behavior Scales
GMD: Gray Matter Densities
iRBD: Idiopathic RBD
LDT: Laterodorsal Tegmental
MGN: Medial Geniculate Nucleus
mSN: Medial Septal Nucleus
MSNs: Medium Spiny Neurons
MVN: Medial Vestibular Nucleus
nAChR: Nicotinic Acetylcholine Receptor
nhlDBB: Nucleus of the Horizontal Limb of the Diagonal Band of Broca
nvlDBB: Nucleus of the Vertical Limb of the Diagonal Band of Broca
PD: Parkinson’s Disease
PET: Positron Emission Tomography
PFN: Parafascicular Nucleus
PIGD: Postural Instability and Gait Difficulties
PPN: Pedunculopontine Nucleus
RBD: Rapid eye Movement Sleep Behavior Disorder
SN: Substantia Nigra
SNc: Substantia Nigra Compact
SNr: Substantia Nigra Reticulum
SPECT: Single-photon Emission Computed Tomography
